# Possible Dual Role of Decorin in Abdominal Aortic Aneurysm

**DOI:** 10.1371/journal.pone.0120689

**Published:** 2015-03-17

**Authors:** Koshiro Ueda, Koichi Yoshimura, Osamu Yamashita, Takasuke Harada, Noriyasu Morikage, Kimikazu Hamano

**Affiliations:** 1 Department of Surgery and Clinical Science, Yamaguchi University Graduate School of Medicine, Ube, 755–8505, Japan; 2 Graduate School of Health and Welfare, Yamaguchi Prefectural University, Yamaguchi, 753–8502, Japan; Yokohama City University Graduate School of Medicine, JAPAN

## Abstract

Abdominal aortic aneurysm (AAA) is characterized by chronic inflammation, which leads to pathological remodeling of the extracellular matrix. Decorin, a small leucine-rich repeat proteoglycan, has been suggested to regulate inflammation and stabilize the extracellular matrix. Therefore, the present study investigated the role of decorin in the pathogenesis of AAA. Decorin was localized in the aortic adventitia under normal conditions in both mice and humans. AAA was induced in mice using CaCl_2_ treatment. Initially, decorin protein levels decreased, but as AAA progressed decorin levels increased in all layers. Local administration of exogenous decorin prevented the development of CaCl_2_-induced AAA. However, decorin was highly expressed in the degenerative lesions of human AAA walls, and this expression positively correlated with matrix metalloproteinase (MMP)-9 expression. In cell culture experiments, the addition of decorin inhibited secretion of MMP-9 in vascular smooth muscle cells, but had the opposite effect in macrophages. The results suggest that decorin plays a dual role in AAA. Adventitial decorin in normal aorta may protect against the development of AAA, but macrophages expressing decorin in AAA walls may facilitate the progression of AAA by up-regulating MMP-9 secretion.

## Introduction

Abdominal aortic aneurysm (AAA) is a segmental expansion of the abdominal aorta. AAA is a common, fatal disease that can cause catastrophic aneurysmal ruptures [1]. The prevalence of AAA is estimated to be between 4.0% and 8.9% in older men [2]. Aortic aneurysms were the primary cause of 10,597 deaths and a contributing cause in 17,215 deaths in the United States in 2009 [3,4]. Because most patients with AAA have no symptoms, the main purpose of treatment is to improve prognosis by preventing aneurysmal rupture. Therapeutic options for AAA are currently limited to open or endovascular surgical repair to prevent rupture [5]. An important unmet need in the treatment of AAA is non-surgical approaches, particularly pharmacotherapies [6–8].

AAA is characterized by chronic inflammation and extracellular matrix degradation caused by proteolytic enzymes, such as matrix metalloproteinase (MMP)-9 [6,9]. Although no fundamental cause of AAA has been identified, various proinflammatory mediators are activated in AAA walls, causing the inflammatory responses and shifting the balance of extracellular matrix metabolism towards tissue degradation [8]. Extracellular matrix proteins are major constituents of the vascular wall and can potentially interact with a variety of vascular cells. Extracellular matrix proteins, such as periostin and tenascin C, have been suggested to play important roles in the pathogenesis of AAA [10–12]. However, no study has fully elucidated the significance of other various extracellular matrix proteins in AAA.

Decorin belongs to the family of small leucine-rich proteoglycans (SLRP) thought to play essential roles in vascular biology [13,14]. In particular, decorin is capable of modulating collagen fibrillogenesis, immune responses, and inflammatory responses [14,15]. Decorin is expressed, to some extent, in normal aortic tissues and aneurysm walls. A previous study showed that reduced decorin expression is associated with a high risk of aortic rupture in a mouse model of AAA [16]. These findings led us to hypothesize that decorin plays a crucial role in the pathogenesis of AAA. In the present study, we show that decorin has both tissue-protective and pro-inflammatory properties, depending on the context in which it is expressed.

## Materials and Methods

### Animal experiments

Six-week-old male C57BL/6 mice were purchased from Chiyoda Kaihatsu Co., Ltd. (Tokyo, Japan). The mice were maintained in plastic cages (5 per cage) in a temperature- and humidity-controlled room with a 12-h light/12-h dark cycle. Mice were allowed free access to standard food and water throughout the experiments. We induced AAA in mice with periaortic application of 0.5 M CaCl_2_ as described previously [12,17,18]. More precisely, we treated the infrarenal aorta between the left renal vein and aortic bifurcation with CaCl_2_. In the prevention study, we placed Gelfoam patches (3.5×2×2 mm; Pfizer, New York, NY, USA) in the periaortic space between the left renal vein and aortic bifurcation immediately after CaCl_2_ treatment; the patches were loaded with either 20 (g bovine decorin (Sigma-Aldrich, St. Louis, MO, USA) dissolved in 100 (l phosphate-buffered saline (PBS) (CaCl_2_+decorin, n = 10) or 100 (l PBS (CaCl_2_+PBS, n = 9). Untreated mice served as the control group (Control, n = 6).

For these studies, mice were anesthetized with an intraperitoneal injection of sodium pentobarbital (40 mg/kg) before undergoing laparotomy. The experimental mice were sacrificed with an overdose of sodium pentobarbital (100 mg/kg, intraperitoneal injection) 42 days after CaCl_2_ treatment for the prevention study, or 0, 3, 7, 14, 28, or 42 days after CaCl_2_ treatment for the temporal observation study. Tissue was fixed with whole-body perfusion of 4% paraformaldehyde in PBS at physiological pressure and the abdominal aorta immediately excised, photographed for morphometric analysis, and sections analyzed histologically. Photographs of the aortas were used to determine maximum external aortic diameters.

All experiments were performed in accordance with the Guide for the Care and Use of Laboratory Animals published by the United States National Institutes of Health. All protocols were approved by the Yamaguchi University School of Medicine Animal Experiments Review Board (#31–091).

### Histological and immunohistochemical analyses

For histological analyses, paraffin-embedded sections were stained with hematoxylin and eosin (HE) and elastica-van Gieson (EVG). Sections were also probed with antibodies raised against appropriate antigens for immunohistochemistry as described previously [17,19,20]. We detected decorin by probing sections with anti-mouse decorin antibody (Santa Cruz Biotechnology, Dallas, TX, USA, #sc-22753) and anti-human decorin antibody (R&D Systems, Minneapolis, MN, USA, #MAB143), and detected MMP-9 by probing sections with anti-mouse MMP-9 antibody (R&D Systems, #AF909) and anti-human MMP-9 antibody (Daiichi Fine Chemical, Toyama, Japan, # F-69). We also used anti-human CD68 antibody (Dako, Glostrup, Denmark, #M0876) and anti-human smooth muscle actin antibody (Dako, #M0851). The probed proteins were visualized by the avidin-biotin complex technique using the VECTASTAIN ABC-AP kit (Vector Laboratories, Burlingame, CA, USA) or by indirect immunofluorescence staining using Alexa Fluor 488-conjugated anti-mouse IgG antibody (Molecular Probes, Eugene, OR, USA) and Alexa Fluor 594-conjugated anti-rabbit IgG antibody (Molecular Probes). DAPI (Molecular Probes) was used for nuclear staining.

The total number of infiltrating mononuclear cells was counted in five high-power fields per mouse in HE-stained sections. We also evaluated the degree of medial layer elastin disruption in EVG-stained sections using the grading method reported by Hamblin et al. [21]. Briefly, the degree of elastin degradation was classified as mildly disrupted when only one elastic lamella was disrupted (grade I), moderately disrupted when two elastic layers were broken or disrupted (grade II), highly disrupted when three elastic layers exhibited breakage and/or degradation (grade III), and severely disrupted when all four elastic layers exhibited signs of breakage and/or degradation (grade IV).

### Cell culture experiments

Rat aortic vascular smooth muscle cells (VSMCs) derived from the medial layer of healthy rat aorta were purchased from Cell Applications, Inc (San Diego, CA, USA). VSMCs were maintained in Dulbecco’s modified Eagle’s medium (DMEM) (Invitrogen, Carlsbad, CA, USA) containing 10% fetal bovine serum. Before the experiments, VSMCs were seeded onto laminin-coated plates and serum-starved for 48 h. The starved cells were treated with 100 ng/ml lipopolysaccharide (LPS) (Alexis Biochemicals, San Diego, CA, USA) for 48 h. When indicated, the VSMCs were treated with 0.4, 4, or 40 μg/ml bovine decorin (Sigma-Aldrich) for 24 h prior to LPS administration.

Thioglycolate-elicited peritoneal macrophages were collected from 6-week-old male C57BL/6 mice (Chiyoda Kaihatsu) as described previously [22,23]. Briefly, the mice were injected intraperitoneally with 2 ml of thioglycolate medium (Sigma-Aldrich). After 3 days, cells were harvested by peritoneal lavage with 10 ml PBS. The cells were washed twice with cold PBS, resuspended in RPMI-1640 medium (DS Pharma Biomedical, Osaka, Japan) containing 10% fetal bovine serum, and seeded on gelatin-coated plates. After 24 h, non-adherent cells were removed by washing the cultures with medium. The peritoneal cells were immunostained with anti-mouse Mac3 antibody (BD Biosciences, San Jose, CA, USA, #550292) to identify macrophages. In all cases, the proportion of macrophages was consistently >90%. Before experiments, macrophages were serum-starved for 24 h, followed by treatment with 100 ng/ml LPS for 48 h. When indicated, cells were treated with 0.4, 4, or 40 μg/ml decorin for 24 h prior to LPS administration.

### Gelatin zymography

Gelatin zymography was performed as described previously [17,19]. Briefly, equal volumes of conditioned media were electrophoresed in the presence of 0.2% SDS on a 10% polyacrylamide gel containing gelatin (1 mg/ml) under non-reducing conditions. After electrophoresis, the gels were washed in 2.5% Triton X-100 and incubated at 37°C in developing buffer (50 mM Tris (pH 7.5), 200 mM NaCl, 5 mM CaCl_2_, and 0.02% Briji35). The gels were stained with 0.5% Coomassie brilliant blue R-250 in 40% methanol and 10% acetic acid and the expression of MMP-9 and MMP-2 determined by quantifying bands at appropriate positions on the gel.

### Human aortic samples

We obtained abdominal aortic wall specimens from 47 patients with AAA undergoing open surgical repair. As controls, non-aneurysmal abdominal aortic wall specimens were obtained from four autopsy patients who died of unrelated causes. The aortic tissue specimens were used for protein analyses by western blotting and immunohistochemistry. All patients provided written informed consent in accordance with the principles outlined in the Declaration of Helsinki. Regarding autopsy specimens, written informed consent was obtained from the next of kin for the use of the sample in research. All experimental protocols with human specimens were approved by the Institutional Review Board at Yamaguchi University Hospital (#H24–26).

### Protein isolation and western blotting

Human aortic wall specimens were homogenized in a solution of 25 mM Tris (pH 7.4), 150 mM NaCl, 5 mM EDTA, 10 mM sodium pyrophosphate, 10 mM β-glycerophosphate, 1 mM Na_3_VO_4_, 1 mM phenylmethane sulfonyl fluoride, and 10 μg/ml aprotinin. Proteins were extracted by adding Triton X-100 to a final concentration of 1%. Protein concentrations were determined using a bicinchoninic protein assay kit (BCA kit, Bio-Rad, Hercules, CA, USA).

Western blotting was performed as described previously [17,20]. Briefly, equal amounts of sample protein were loaded onto each lane of an SDS-PAGE gel. Separated proteins were transferred onto polyvinylidene difluoride membranes (Millipore, Bedford, MA, USA) and probed with antibodies for human decorin (R&D Systems, #MAB143), glyceraldehyde 3-phosphate dehydrogenase (GAPDH) (Millipore, # MAB374), human MMP-9 (Daiichi Fine Chemical, # F-69), and human transforming growth factor (TGF)-β 1 (Santa Cruz Biotechnology, #sc-52893).

### Enzyme-linked immunosorbent assay (ELISA)

The concentration of TGF-β in conditioned media was quantified by a sandwich enzyme immunoassay technique using the mouse/rat/porcine TGF-β 1 ELISA Kit (R&D Systems, #SMB100) according to the manufacturer’s instructions.

### Statistical analysis

Data are expressed as the mean ± standard deviation (SD). Statistical analyses were performed with Prism 5.0d software (GraphPad Software, La Jolla, CA, USA). The unpaired *t*-test or analysis of variance (ANOVA) was used for comparisons with Bonferroni post-test correction. Associations between continuous variables were assessed by the Pearson correlation coefficient test. A p-value <0.05 was considered significant.

## Results

### Temporal pattern of decorin protein levels during AAA development in mice

**Fig 1:**
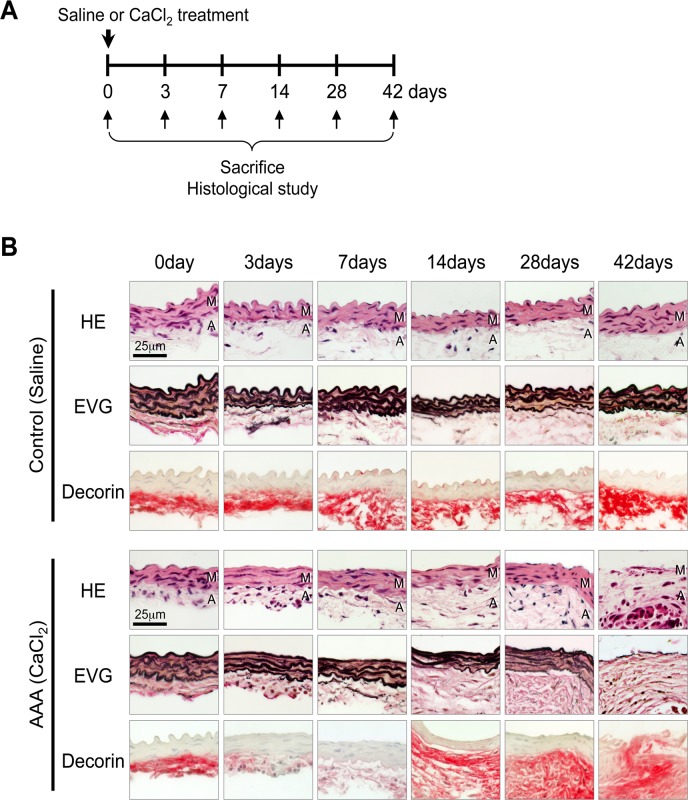
Temporal pattern of decorin protein levels during AAA development in mice. (**A**) A mouse model of AAA was induced by periaortic application of CaCl_2_. Saline application was used as a control. The mice were sacrificed 0, 3, 7, 14, 28, or 42 days after CaCl_2_ treatment. (**B**) Representative images showing aortic walls stained with hematoxylin and eosin (HE), elastica van-Gieson (EVG), or an antibody against decorin at the indicated time points after the application of saline (Control) or CaCl_2_ (AAA induction). The luminal surface is oriented toward the top of each panel. HE and EVG stains depict cell nuclei (blue-black) and the elastin network (black), respectively. Localization of decorin is indicated by red staining. M: media, A: adventitia.

First, we analyzed a mouse model of AAA to evaluate decorin protein levels during the initiation, development, and progression of AAA. The mouse model was created by periaortic application of CaCl_2_ in the infrarenal aorta (Fig. 1A). After 3 days, the CaCl_2_ treatment induced infiltration of inflammatory cells. From 3 to 14 days, the elastic lamellae began to exhibit straightening and fragmentation. Inflammatory cell infiltration and the disruption of elastic layers continued and gradually increased up to 42 days (Fig. 1B). Consequently, 28 and 42 days after CaCl_2_ treatment, the infrarenal aortas of AAA mice exhibited significantly larger maximum diameters than the controls, which was consistent with our previous results [12].

In the control mice, decorin deposition was observed in the adventitia and periaortic tissues, and protein levels were similar before and after saline treatment. In the AAA mice, adventitial decorin was severely diminished 3 and 7 days after CaCl_2_ treatment, but by 14 days the decorin protein levels returned to nearly basal levels and began to spread to periaortic tissues. At 42 days, we observed decorin deposition in all layers, including the thin aortic media, where we observed marked increases in inflammatory cell infiltration and elastic lamellae destruction (Fig. 1B). These results indicate that decorin protein is down-regulated at the initiation of AAA. However, during the progression of AAA, decorin protein was inversely up-regulated and spread to the aortic walls, where active inflammation caused destruction of the elastic layers.

### Effect of decorin administration on the development of AAA in mice

**Fig 2:**
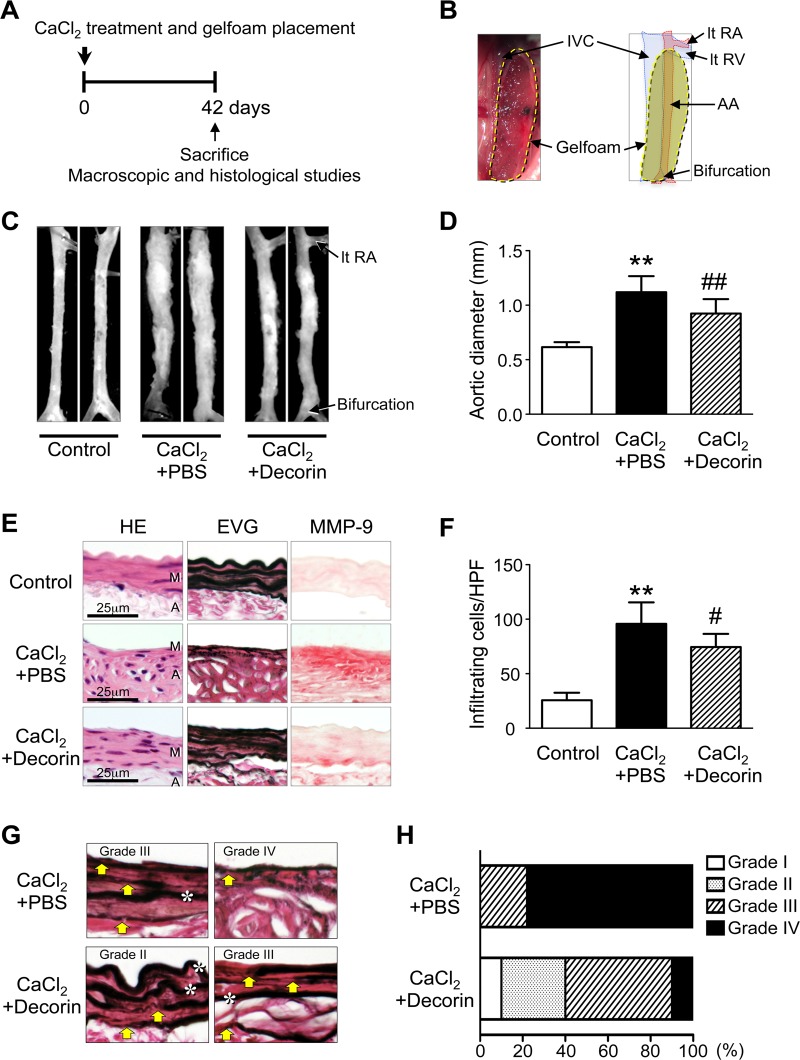
Effect of decorin administration on AAA development in mice. (**A-B**) Immediately after CaCl_2_ treatment, Gelfoam patches loaded with PBS (CaCl_2_+PBS, n = 9) or 20 (g decorin (CaCl_2_+decorin, n = 10) were placed into the periaortic spaces between the left renal vein (lt RV) and bifurcation. AA: abdominal aorta, IVC: inferior vena cava, lt RA: left renal artery. Untreated mice were used as controls (Control, n = 6). The mice were sacrificed 42 days after CaCl_2_ treatment. (**C**) Representative photographs show aortas 42 days after CaCl_2_ treatment. (**D**) Quantitative analysis of the maximum external diameters of abdominal aortas. Data are mean ± SD. **p<0.01 compared to Control; ##*p*<0.01 compared to CaCl_2_+PBS. (**E**) Representative histological and immunohistochemical stains of abdominal aorta specimens from mice treated as indicated. HE and EVG stains depict cell nuclei (blue-black) and elastin network (black), respectively. The localization of MMP-9 is indicated with red staining. M: media, A: adventitia. (**F**) Infiltrating mononuclear cells were counted in five high-power fields. Data are mean ± SD. **p<0.01 compared to Control; #*p*<0.05 compared to CaCl_2_+PBS. (**G-H**) The degree of medial layer elastin disruption was graded as mild (grade I), moderate (grade II), high (grade III), or severe (grade IV) based on EVG-stained sections. Yellow arrows indicate disruption of elastic lamellae. White asterisks indicate preserved elastic lamellae.

To clarify the relationship between the initial decline in decorin and the initiation of AAA, we investigated whether local application of exogenous decorin inhibits pathological remodeling of the aortic wall in the AAA mouse (Fig. 2A). We applied a decorin protein solution to Gelfoam, a biodegradable extracellular matrix preparation that provides local delivery of proteins of interest [12,24], and implanted the Gelfoam patches (CaCl_2_+decorin or CaCl_2_+PBS) in the periaortic space of mice immediately after CaCl_2_ treatment (Fig. 2B). According to Kuhn B. et al. [24], this delivery system could theoretically enable continuous release of decorin during the experimental period and deliver decorin to the aortic media in mice. Forty-two days after treatment, aortas from the CaCl_2_+PBS group exhibited a significant increase in aortic diameter compared to untreated aortas (Control group) (Fig. 2C-D), which was similar to our previous results [11,12,17,25]. The CaCl_2_+PBS group also had marked inflammatory cell infiltration in all layers, and the morphology of the elastic lamellae in the aortic media appeared straightened and fragmented. We found abundant MMP-9 expression in the media and adventitia, in addition to cellular infiltration and medial destruction, in the CaCl_2_+PBS group (Fig. 2E-F). Interestingly, aortas from the CaCl_2_+decorin group had significantly smaller diameters than those from the CaCl_2_+PBS group (Fig. 2C-D). Histological analyses clearly demonstrated that aortas from the CaCl_2_+decorin group had fewer infiltrating mononuclear cells, more preserved elastic lamellae morphology, and lower MMP-9 protein levels compared to aortas from the CaCl_2_+PBS group (Fig. 2E-F).

Because excessive elastolysis mediated by MMP-9 is considered a critical step in aneurysm development [9,20], we determined the degree of medial elastin disruption in the CaCl_2_+PBS and CaCl_2_+decorin groups (Fig. 2G). Most (78%) of the aortas in the CaCl_2_+PBS group had severe elastin disruption (grade IV). In contrast, only 10% of the aortas in the CaCl_2_+decorin group had grade IV disruption. Most of the aortas in the CaCl_2_+decorin group exhibited moderate (grade II, 30%) or high elastin disruption (grade III, 50%) (Fig. 2H). These findings indicate that the initial decline in adventitial decorin potentially contributes to the development of CaCl_2_-induced AAA. Preventing this decline in decorin by applying exogenous decorin resulted in significant suppression of AAA development, probably by inhibiting MMP-9-mediated elastin disruption.

### Role of decorin in VSMC secretion of MMP-9

**Fig 3:**
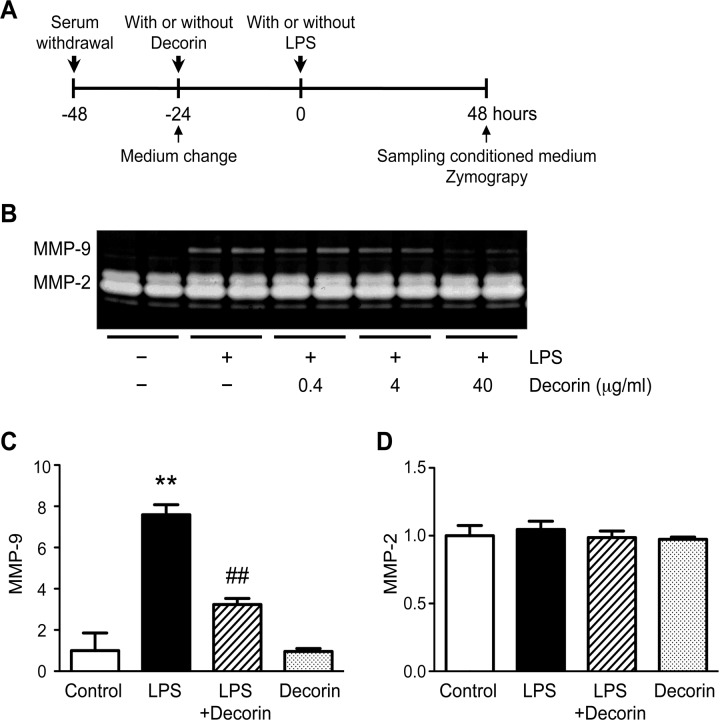
Role of decorin in VSMC secretion of MMP-9. (**A**) Cultured rat vascular smooth muscle cells (VSMCs) were pre-treated with or without 0.4, 4, or 40 μg/ml decorin, then stimulated with or without 100 ng/ml lipopolysaccharide (LPS) for 48 h. Protein levels of MMP-9 and MMP-2 in the conditioned media were determined by gelatin zymography. (**B**) Representative results are shown. (**C-D**) Cultured rat VSMCs were pre-treated with or without 40 μg/ml decorin, then stimulated with or without 100 ng/ml LPS for 48 h. Quantitative analyses for MMP-9 (**C**) and MMP-2 (**D**) are shown. Data are mean ± SD. **p<0.01 compared to Control; ##*p*<0.01 compared to LPS.

Because our *in vivo* findings suggested that decorin inhibits MMP-9 expression in the aortic wall, we investigated whether decorin negatively regulates MMP-9 protein secretion in VSMCs, one of the major cell types of the aortic wall (Fig. 3A). Under basal conditions, MMP-2 was readily detectable in the conditioned media of VSMCs, but MMP-9 was nearly undetectable. However, the VSMCs secreted appreciable levels of MMP-9 in response to LPS treatment. Interestingly, this LPS-induced up-regulation of MMP-9 secretion was abrogated by pre-treatment with exogenous decorin in a dose-dependent manner (Fig. 3B-C). Neither LPS nor decorin affected the secretion of MMP-2, which suggests that cell viability was preserved during the experiments (Fig. 3D). These data demonstrate that decorin plays a role in protecting VSMCs from inflammatory insults by inhibiting the up-regulation of MMP-9.

### Expression of decorin in human AAA

**Fig 4:**
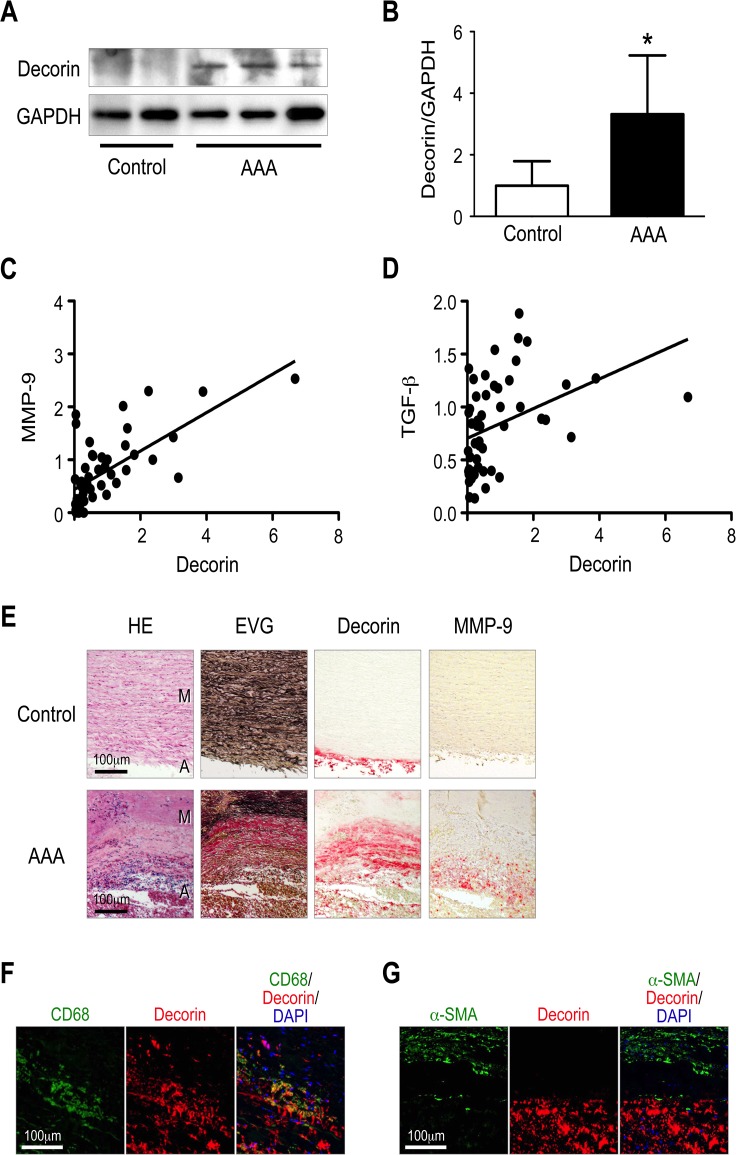
Expression of decorin in human AAA. (**A-B**) Protein samples were obtained from the aortic walls of human AAA specimens (n = 47) and non-aneurysmal specimens (Control, n = 4). Representative results from western blot detection of decorin are shown (**A**) with the corresponding quantitative analysis (**B**). GAPDH served as an internal control. Data are mean ± SD. *p<0.05 compared to Control. (**C**) The correlation between the protein levels of decorin and MMP-9 was examined and the quantitative analysis is shown (Pearson r = 0.68, n = 51, *p*< 0.001). (**D**) The correlation between the protein levels of decorin and TGF-β was examined and the quantitative analysis is shown (Pearson r = 0.39, n = 51, *p*< 0.01). (**E**) Representative histological and immunohistochemical stains are shown for human AAA wall specimens. The luminal surface is oriented toward the top of each panel. HE and EVG stains depict cell nuclei (blue-black) and elastin network (black), respectively. The localization of decorin and MMP-9 is indicated by red staining. M: media, A: adventitia. (**F-G**) Representative images of immunofluorescence staining for decorin (red) and CD68 (macrophage marker, green, **F**) or α-smooth muscle actin (α-SMA) (smooth muscle cell marker, green, **G**). Yellow in the merged images indicates overlapping localization of the red and green signals.

To determine whether these findings are applicable to humans, we examined decorin protein levels in human AAA specimens. Because we obtained the human AAA specimens from patients who had undergone open surgery, the specimens represented progressed lesions rather than initial lesions. Thus, these lesions most likely correspond to the mouse AAA specimens 42 days after CaCl_2_ treatment. As expected, decorin expression was greatly elevated in the human AAA walls compared to non-aneurysmal aortic walls (Controls) (Fig. 4A-B). MMP-9 expression was also highly elevated in the human AAA walls, as reported previously [26,27]. Interestingly, decorin protein levels positively correlated with MMP-9 protein levels in human specimens (Fig. 4C). TGF-β protein, a possible stabilizer of extracellular matrix in AAA [28] also positively correlated with decorin protein levels in human aortic specimens (Fig. 4D).

Next, we determined the tissue localization of decorin and MMP-9 and analyzed their association with pathological tissue architecture in human AAA walls. The AAA walls typically exhibited cellular infiltration, fragmentation, and loss of elastic lamellae. Although decorin and MMP-9 did not strictly co-localize, both decorin and MMP-9 were found mainly in the media and adventitia, frequently accompanied by severe inflammation and tissue destruction (Fig. 4E). Immunofluorescence staining also showed that decorin largely colocalized with CD68^+^ macrophages in the media and adventitia, but not with α-SMA^+^ smooth muscle cells (Fig. 4F-G). In contrast to AAA walls, non-aneurysmal aortic walls exhibited decorin deposition in the adventitia, not the media, and they lacked MMP-9 expression, which is consistent with our observations in control mice. Taken together, our observations in humans and mice suggest that decorin plays a protective role in AAA development. However, in progressed AAA walls, decorin positively correlates with MMP-9, suggesting that decorin plays a stimulatory role in AAA progression.

### Role of decorin in macrophage secretion of MMP-9

**Fig 5:**
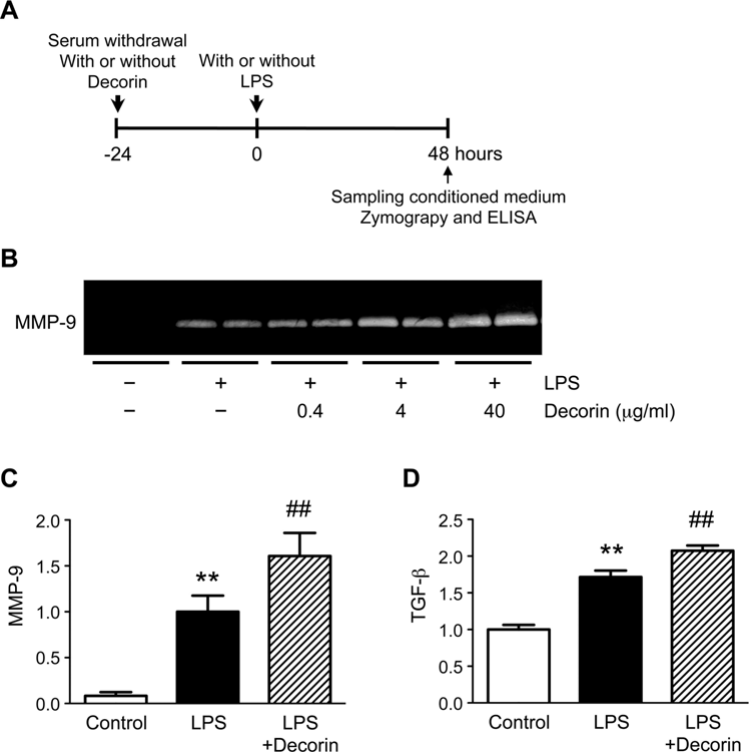
Role of decorin in macrophage secretion of MMP-9. (**A**) Cultured mouse macrophages were pre-treated with or without 0.4, 4, or 40 μg/ml decorin, then stimulated with or without 100 ng/ml lipopolysaccharide (LPS) for 48 h. (**B**) MMP-9 protein levels in the conditioned media were determined by gelatin zymography. Representative results are shown. (**C-D**) Cultured mouse macrophages were pre-treated with or without 40 μg/ml decorin, then stimulated with or without 100 ng/ml LPS for 48 h. Quantitative analyses of MMP-9 (**C**) and TGF-β (**D**) are shown. TGF-β protein levels in the conditioned media were determined by enzyme-linked immunosorbent assay (ELISA). Data are mean ± SD. **p<0.01 compared to Control; ##*p*<0.01 compared to LPS.

To understand the conflicting findings regarding the role of decorin in aortic walls, we investigated whether decorin positively regulates MMP-9 secretion in macrophages, the major inflammatory cell-type secreting MMP-9 in AAA walls [9,17] (Fig. 5A). Under basal conditions, MMP-9 was nearly undetectable in the conditioned media from cultured mouse macrophages. In response to LPS treatment, macrophages secreted detectable levels of MMP-9. Intriguingly, pre-treatment with exogenous decorin resulted in a dose-dependent increase in LPS-induced MMP-9 secretion (Fig. 5B-C). In addition, LPS treatment increased macrophage secretion of TGF-β, and pre-treatment with exogenous decorin further enhanced this secretion (Fig. 5D).

The data demonstrate that decorin stimulates macrophage secretion of MMP-9 and inhibits VSMC secretion of MMP-9. Thus, our findings reveal that decorin has opposite effects on MMP-9 secretion depending on the cell type.

## Discussion

This study is the first to demonstrate that adventitial decorin protein initially decreases in a CaCl_2_-induced AAA mouse model and that local administration of decorin can inhibit the development of AAA. These findings indicate that a decline in adventitial decorin may result in the initiation of AAA. Decorin expression in adventitial cells may indirectly result in protection of the aortic media against inflammatory insults by unknown mechanisms. However, we presented another possibility; decorin, which is released from the adventitia of normal and non-aneurysmal aortic walls, can reach the media, affect medial VSMCs, and directly protect the media from proteolytic degradation. Consistent with our results, reduced expression of decorin in the adventitia was associated with a high risk of aortic rupture in another mouse model of AAA induced by angiotensin II [16]. Among human aneurysmal diseases, deficient decorin expression has been associated with lethal forms of Marfan’s syndrome [29] and aortic dissection [30,31].

Decorin is characterized by its interactions with a multitude of structural components within the extracellular matrix, particularly collagen and elastin fibers, and it is important for the regulation of collagen fibrillogenesis. Decorin may be associated with elastinogenesis [14,32]. Similar to other SLRPs, decorin can help protect collagen fibrils from cleavage by collagenases [33]. The data indicate that stabilization of collagen and elastin fibers by decorin may contribute to the prevention of tissue destruction in our AAA model. Another possibility is that decorin inhibits AAA development by suppressing the expression of pro-inflammatory molecules and cellular infiltration. In the present study, we showed that local administration of decorin results in a significant decrease in inflammatory cell infiltration and MMP-9 protein levels. We also demonstrated that decorin can negatively affect MMP-9 secretion in cultured VSMCs. Systemic over-expression of decorin was previously reported to reduce macrophage infiltration and gelatinase activity in a mouse model of atherosclerosis [34]. Administration of decorin antagonized multiple receptor tyrosine kinases (e.g., Met), subsequently leading to the down-regulation of MMP-9 in the context of tumor angiogenesis [35]. In addition, decorin deficiency results in the up-regulation of MMP-9 in mouse fetal membranes at embryonic day 18 [36].

In contrast, decorin was recently shown to induce pro-inflammatory signaling, thereby linking innate immunity, inflammation, and tumorigenesis [15,37,38]. Decorin was reported to interact with toll-like receptor (TLR) 2 and TLR4 and stimulate the expression of cytokines, primarily pro-inflammatory tumor necrosis factor (TNF)-α and pro-interleukin (IL)-1β, by activating nuclear factor (NF)-κB in macrophages. Decorin reportedly augments the expression of MMP-9 in cancer cell lines [39]. Decorin has also been shown to enhance the effects of LPS, a major ligand for TLR4, by signaling through TLR2 [38]. Similarly, in this study, we demonstrated that decorin enhances LPS-induced MMP-9 secretion in macrophages. In addition, decorin blocked the binding of TGF-β to its receptor, which reduced the production of anti-inflammatory cytokine IL-10 [15,38]. Thus, these data indicate that decorin signaling stimulates inflammatory responses under certain conditions. In a mouse model of contact dermatitis, decorin deficiency resulted in the reduced expression of chemokines, including KC/CXCL-1 and MCP-1/CCL2, and attenuated leukocyte recruitment [40]. In another study, decorin deficiency inhibited the inflammatory reaction in a mouse model of allergen-induced asthma [41]. Therefore, decorin appears to be a double-edged sword with potentially both pro-inflammatory and anti-inflammatory functions.

We showed that decorin protein levels are up-regulated in aneurysmal tissues affected by AAA. Other studies have shown that decorin is up-regulated in thoracic aortic aneurysms and intracranial aneurysms [42–44]. We also found that decorin is expressed in abundance in progressed AAA lesions in both mice and humans, and its expression is accompanied by macrophage infiltration, tissue damage, and elevated MMP-9 expression. Our findings strongly suggest that, in destructive AAA lesions, decorin is expressed in macrophages and acts as a pro-inflammatory mediator by enhancing MMP-9 production in macrophages. In addition, decorin has been reported to sequester TGF–β, a stabilizer of the extracellular matrix, and to inhibit its activity, which may accelerate the development of AAA [14]. TGF–β has been shown to stimulate collagen and elastin production, which stabilizes pre-existing AAA induced by angiotensin II in mice [28,45]. Taken together, these findings strongly suggest that decorin can act as an accelerator of tissue destruction under inflammatory conditions. On the other hand, we cannot rule out the possibility that decorin is up-regulated for the purpose of resolving inflammation in AAA. Indeed, our results suggest that decorin positively regulates TGF–β protein levels in macrophages, even in the destructive AAA lesions. However, to date, little is known about the mechanisms regulating the balance between these opposing functions of decorin.

Because decorin potentially has two opposing functions, it may be difficult to use it as a therapeutic target in human AAA. In the progressed stages of AAA, decorin treatments may cause further progression or rupture by up-regulating MMP-9 in macrophages. However, our data indicate that a strategy that preserves or enhances the adventitial expression of decorin may be a promising preventive treatment for individuals at high risk of AAA, and possibly for patients in the initial stages of AAA. Supporting this notion, statins (3-hydroxy-3-methylglutaryl-coenzyme reductase inhibitors), which are commonly used to treat patients with hypercholesterolemia, were reported to increase decorin expression in aortic tissue [46]. In addition, decorin has advantageous properties as a biomarker, as it preferentially accumulates in inflamed and disrupted regions and is released into the circulation. Serum decorin levels have been recognized as a potential biomarker in patients with acute ischemic stroke or esophageal squamous cell carcinoma [47,48]. Thus, decorin may also serve as a useful marker for detecting the active stages of AAA progression.

## Conclusions

Our data demonstrate that adventitial decorin in normal aorta plays a protective role against the development of AAA, but decorin expression in AAA walls may accelerate AAA progression. Thus, this work provides new insights into the dual role of decorin in the molecular pathogenesis of AAA. Decorin may represent a therapeutic target for preventing AAA, and it may serve as a biomarker of AAA progression.
